# Poly-L-Lysine functionalised MWCNT-rGO nanosheets based 3-d hybrid structure for femtomolar level cholesterol detection using cantilever based sensing platform

**DOI:** 10.1038/s41598-019-40259-5

**Published:** 2019-03-06

**Authors:** Aviru Kumar Basu, Amar Nath Sah, Asima Pradhan, Shantanu Bhattacharya

**Affiliations:** 1 Design programme, Indian Institute of Technology, Kanpur, U.P. 208016 India; 2 Microsystems Fabrication Laboratory, Department of Mechanical Engineering, Indian Institute of Technology, Kanpur, U.P. 208016 India; 3 Biophotonics Laboratory, Department of Physics, Indian Institute of Technology, Kanpur, U.P. 208016 India; 4 Department of Biological Sciences and Bioengineering, Indian Institute of Technology, Kanpur, U.P. 208016 India; 5 Department of Physics, Indian Institute of Technology, Kanpur, U.P. 208016 India

## Abstract

In this work we have developed a novel rGO-MWCNT (reduced graphene oxide-multiwalled carbon nanotube) nanocomposite material with Poly-L-Lysine functionalization which can be used for detection of biomolecules with enhanced sensitivity. The reduced GO sheets are found to play a major role as a connector and helps in the assembly of bundles of carbon nanotubes (CNTs) which may sometime play a role of upstanding nanostructures. The overall composite structure is further fully functionalized resulting in an overall high density of amino groups that can be used to capture biomolecules. The sensitivity of the as synthesized film is tested by the oxidation of cholesterol through cholesterol oxidase enzyme that is biochemically immobilized over these composite films. The test for the immobilization density of the novel films are carried out by mounting these films on sensitive thin section static micro/nano-cantilever platforms. The platforms have capability to measure cholesterol traces in blood upto an extent of 100 femto molar through deflection /bending of the cantilevers due to surface reaction. The films developed show a promise of high immobilization density which is further confirmed through fluorescence studies using FITC labeling of functionalized MWCNT-PLL and rGO-PLL films respectively.

## Introduction

Graphene and its different manifestations have always attracted a lot of attention worldwide among the sensor community^1,2^. The principle reasons for graphene family to be so well explored is its exceptional mechanical, thermal, electrical and architectural properties^3–5^. Of significant mention is its high surface area and also planarity which makes it the obvious choice for serving as a functional material and the backbone of many of the nano-composite forms of graphene family^3^. Examples are the rigorous use of reduced graphene oxide sheets for fabrication of electronic and sensor devices like field effect transistors, electromechanical resonators, a battery of sensors for sensing biochemical, chemical; and other gaseous analytes etc.^4–7^. The graphene paper which was developed a few years back has already exhibited excellent cell adhesion and growth with good biocompatibility of the graphene material etc.^8^. Some recent examples have been the increased use of reduced graphene oxide for bio-applications, such as preferential planar binding of single stranded nucleic acid structures on the surfaces that can be used for sensitive hybridization, expression monitoring, transcription profiling ^9–11^, hybrid devices fabricated with live bacteria etc.^12^. Dai *et al*. used branched polyethylene glycol synthesized rGO sheets to bind to aromatic water insoluble drugs so that they could be delivered *in-vivo*^13^.

Rolled graphene sheets otherwise known as Single-walled carbon nanotubes (SWNTs), have shown a lot of utility prior to the planar graphene structure could be isolated mostly due to their superior properties and a wide range of applications obtained through the derivatization of the sides and ends of the nanotubes^14–16^. SWNTs have exhibited wide ranging potential in several biological applications like single molecule sensing, nanoscale targetted drug delivery, tissue regeneration etc.^17,18^. Due to the high surface area of CNTs good chemical stability and more number of poly-aromatic structures, CNTs can adsorb or conjugate with a variety of biomolecules like proteins, drugs, antibodies, DNA, enzyme etc.^19^. Poly-L-Lysine functionalized CNT have been reported to exhibit a high affinity to biomoelcular attachment^20^. Similarly, GO has also been functionalized through PLL to formulate water soluble composites that could perform sensitive detection of H_2_O_2_^21^. Since the Cholesterol-Oxidase in presence of Cholesterol also generates a peroxide reaction therefore it is prudent to utilize the planarity of r-GO substrates and the high density structure of the CNT sidewalls in conjunction while both are outlaid in composite form. The surface area available for this film and the active site density may be multi-folds due to the utilization of the planar 2-D structure as well as the 3-D outspread from the binding planar structure of the r-GO films. Due to the extraordinary high density of the binding sites such films may make possible extremely trace levels of bimolecular species to be detected. The recognition element is bound to this architecture through the PLL functionalization technique. It may also be prudent to mention that the limit of detection of such films may be carried over platforms which are extremely sensitive so that even a trace molarity of the analyte in question do not go undetected once in the presence of the immobilized recognition element.

rGO sheets, have an overall high surface area needing to be formulated very carefully through proper interlayer separation. Otherwise, they may pile up or irreversibly agglomerate to form graphite through the out of plane vander Waals forces or π-π stacking. In this work we have developed a mechanism wherein we have utilized GO sheets as the base layer and used the planar architecture of the rGO to further top mount bundles of MWCNTS. rGO-MWCNT hybrids which are formed by Vander Wall forces or π-π stacking. GO contains π-conjugated aromatic domains in its basal planes and it can interact with the CNTs via π-π interaction. The top mounted CNTs provide resistance to piling thus keeping the base well spread and proper coverage of the whole surface area of the cantilever over which they are spread. The functionalized rGO-CNTs composites have been tested for their efficacy to detect trace concentrations of bio-analytes by using sensitive SU-8 micro-nanocantilevers and studying their deflection by tracing a beam of laser reflected from the top surface of the cantilever surface. The cantilevers as proposed by Stoney *et al*. earlier^22–24^ bend due to a change in the surface energy through binding kinetics of the bio-analyte. While the functionalized films are seen to show maximum deflection, the functionalized planar rGO sheets or functionalized CNT as immobilized on the cantilever surface do not show the same limit of detection. This gives a comparison of the newly fabricated films with respect to standalone functionalized rGO or functionalized CNT films formulated on the surface of the cantilevers. As compared to some of the other functionalization approaches demonstrating immobilization the approach reported in this paper demonstrate a higher level of immobilization density and increased LOD on SU-8 cantilever surface making the cantilevers better in sensitivity and faster in response.

**Figure 1:**
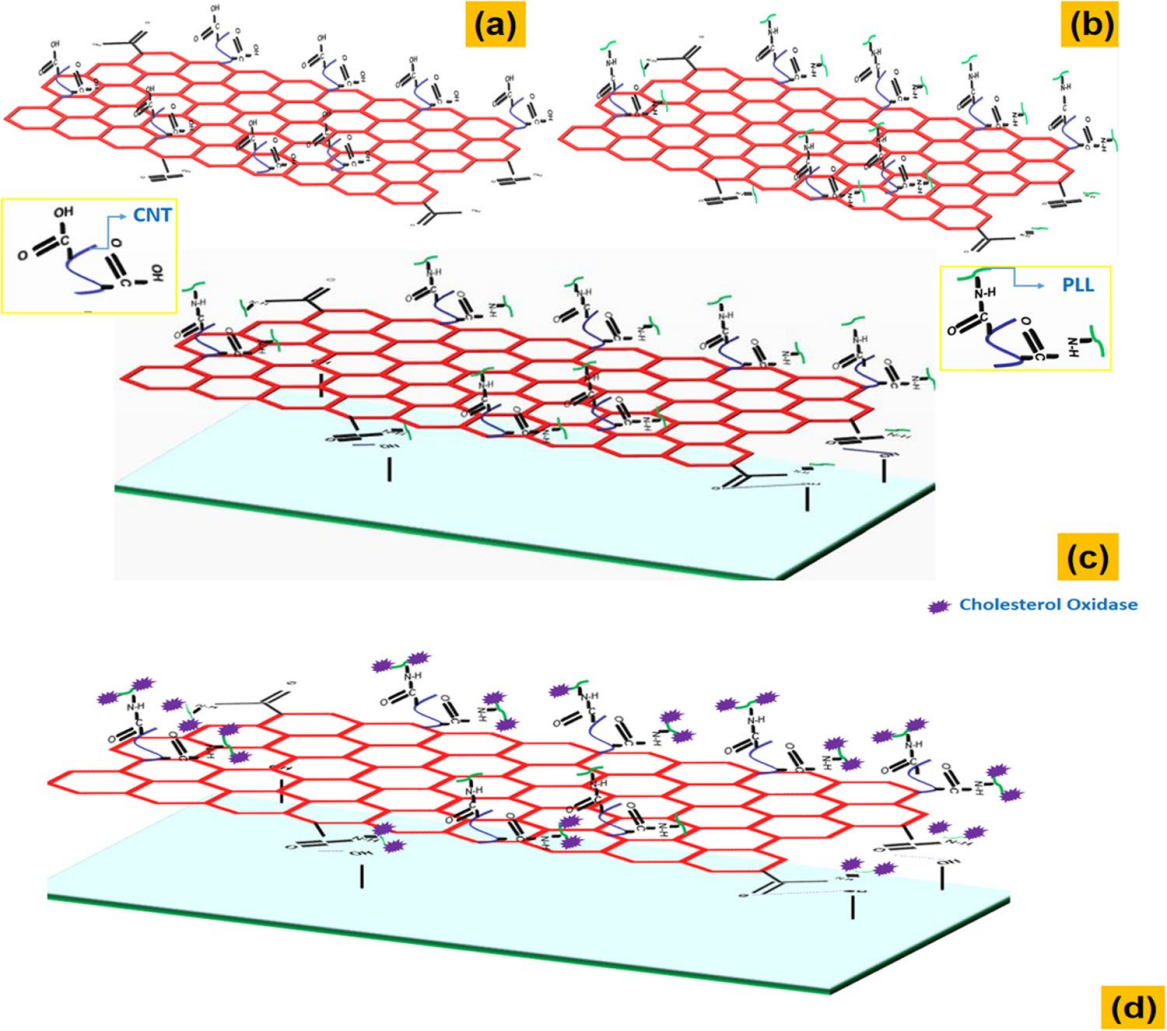
(**a**) COOH functionalized MWCNT attachment on the GO sheets. (**b**) Poly-l-lysine attachment on the GO and CNTs. (**c**) rGO-MWCNT-PLL attachment on the SU-8 surface. (**d**) Cholesterol Oxidase immobilization on the rGO-MWCNT-PLL film.

A schematic representation of rGO-MWCNT-PLL composite formation is shown below in Fig. 1. The material is synthesized as mentioned in methods. The material gets easily attached on the surface of SU-8 polymer due to weak hydrogen bonding and forces of electrostatic attraction with respect to the present hydroxyl moieties on the surface of plasma treated SU-8 polymer. As per the characterization results and from fluorescent studies, we have observed free amine groups to be present or attached to the surface of r-GO films as composites. An abundance of free amine groups on the CNTs and GO sheets play a very critical role for enhancing the sensitivity and lowering the limit of detection for cholesterol molecules.

After the coating of the micro-cantilever surfaces with rGO-MWCNT-PLL nano-composite films, the cantilevers are immobilized with the cholesterol oxidase enzyme (1 mg/ml) through amine linkages and are incubated overnight at 4 °C. The rGO-MWCNT-PLL nano-composite film is linked to the cholesterol oxidase enzyme, creating a bio-sensing platform for cholesterol detection with higher sensitivity and an overall lower limit of detection. The linking is primarily due to amine linkages of the PLL with the cholesterol oxidase enzyme. Figure 1(a–d) show the whole immobilization mechanism on 3-D hybrid structures of the rGO-MWCNT-PLL nano-composite films. The cholesterol oxidase enzyme catalyzes the oxidation process of cholesterol into the products H_2_O_2_ and Cholest-4-en-3-one molecule. The enzymatic reaction with cholesterol oxidase enzyme as a receptor is shown in equation (1) below. The enhanced cantilever deflection curves which are obtained are shown in comparative study of deflection of cantilever with immobilized films section. This is primarily due to more number of cholesterol molecules binding to the cholesterol oxidase enzyme attachment on the rGO-MWCNT-PLL surface.

1 $${\rm{Cholesterol}}+{{\rm{O}}}_{{\rm{2}}}({\rm{from}}\,{\rm{Cholesterol}}\,{\rm{oxidase}})\to {\rm{Cholest}} \mbox{-} {\rm{4}} \mbox{-} {\rm{en}} \mbox{-} {\rm{3}} \mbox{-} {\rm{one}}+{{\rm{H}}}_{{\rm{2}}}{{\rm{O}}}_{{\rm{2}}}$$

**Figure 2:**
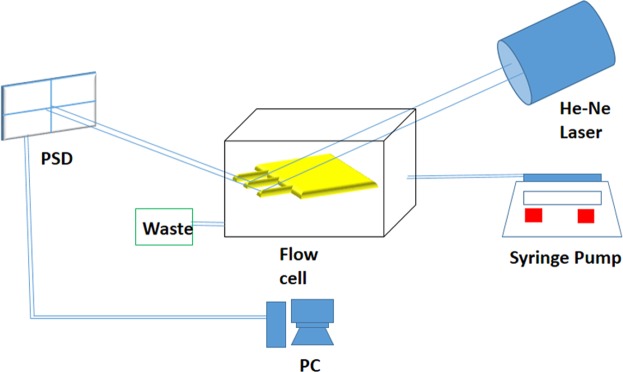
Schematic for Cholesterol Detection on Cantilever.

The cantilevers were observed for small deflections using optical methods wherein an Optical Beam spot focused on the cantilever surface was traced as the static cantilever deflection proceeded due to top loading of the Cholesterol oxidase. The beam tracing was carried out through a high speed EM-CCD camera (Andor) and He-Ne laser of 632 nm wavelength (Thorlabs) trace deflections in the range of tens of nanometers were recorded through image processing algorithm shown in Fig. 2. Multiple observations were recorded for all the four different cantilevers and differential readings indicated a greater deflection. Two spots from the laser beams were focused on the cantilevers, each on the following combinations PLL coated and PLL functionalized CNT coated, PLL coated and PLL functionalized r-GO coated, PLL functionalized CNT coated and PLL functionalized composite coated, PLL functionalized CNT coated and PLL functionalized composite coated etc. Each comparison was duely recorded and used as baseline for obtaining the overall cholesterol detection sensitivity and LOD for the system. There are many situations where clinical needs dictate femtomolar level of analytes to be detected. The system in description can serve as an important tool for carrying out trace level measurements in such situations.

## Results and Discussion

### Material Characterization results

A thorough investigation of the sensing material has been carried out using different types of material characterization techniques.

**Figure 3:**
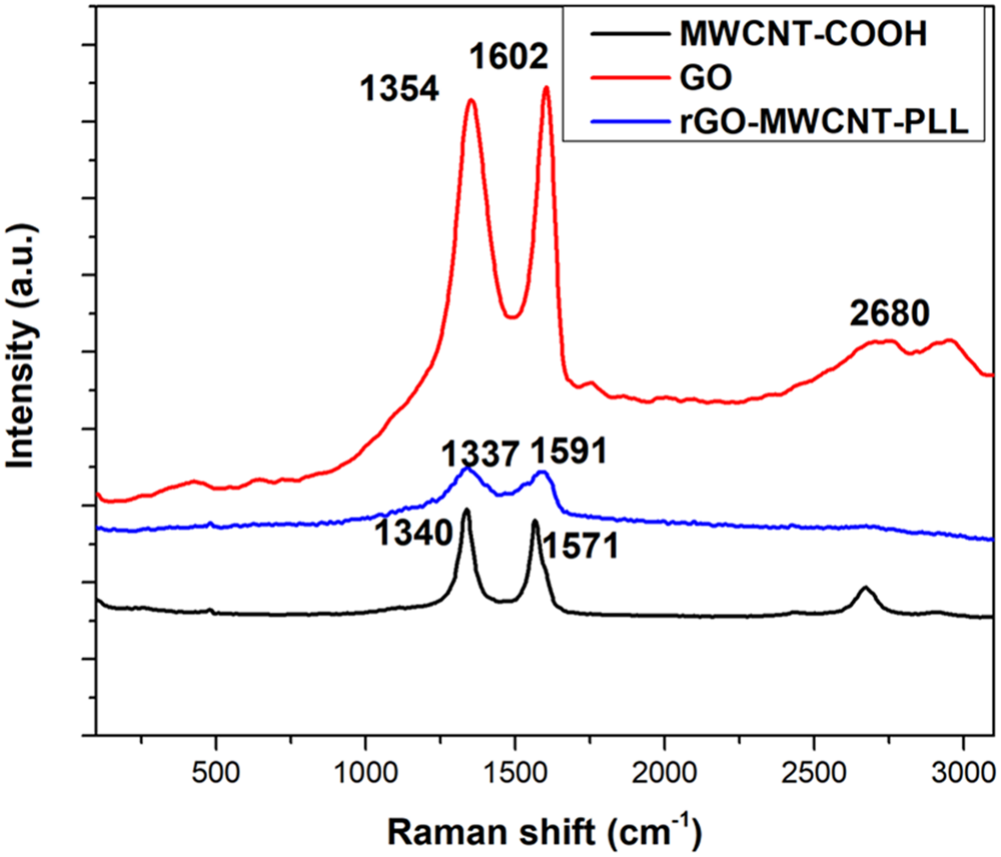
Raman spectra of GO. MWCNT-COOH and rGO-MWCNT-PLL nanocomposite.

Figure 3 shows the Raman Spectra of GO, MWCNT-COOH and rGO-MWCNT-PLL nano-composites etc. As can be observed from the figure shifting has occurred for the D and G band of the nano-composite to the lower frequency compared to Graphene Oxide. The D band has shifted from 1354 to 1337 cm^−1^ and the G band has shifted from 1602 to 1591 cm^−1^ which indicates graphene oxide reduced to graphene. The shift has also occurred for the nano-composite compared to the peaks of MWCNT-COOH at 1340 and 1571 cm^−1^ which proves that there is substantial interaction between the nanotubes, the pol-l-lysine and the GO layers. It can be also observed that the 2D band of GO and CNT are no more visible in the nano-composite material. This is primarily due to the attachment of CNT on the GO layers, as well as due to the attachment of free hanging chains of amino groups on the walls of CNT and the GO sheets.

**Figure 4:**
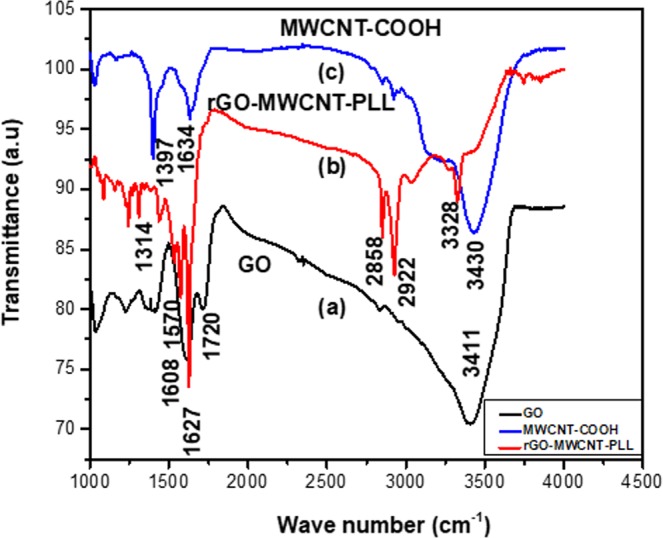
FTIR spectra of GO (**a**), rGO-MWCNT-PLL composite (**b**), and MWCNT-COOH (**c**).

Figure 4 shows the FTIR spectra of GO (Fig. 4a), rGO-MWCNT-PLL (Fig. 4b), MWCNT-COOH (Fig. 4c). From Fig. 4b, it can be clearly seen that there is formation of amine groups which is shown through the response coming from the vibration of amide group I at 1627 cm^−1^ and amide group II at 1570 cm^−1^ and free amine groups at 3328 cm^−1^ respectively. The peak due to C-N stretch mode can be clearly observed^25^ at 1314 cm^−1^. From Fig. 4a,c observations are made about the presence of C=O carbonyl group at 1720 cm^−1^ and 1634 cm^−1^, C=C groups at 1608 cm^−1^ and 1397 cm^−1^, O-H groups due to stretching mode of water at 3430 cm^−1^ and 3411 cm^−1^ respectively. The peaks of O-H groups completely vanish in the composite due to attachments of CNT on the GO surfaces (Fig. 4b), as well as the C=O groups gets shifted from 1720 cm^−1^ for GO to 1627 cm^−1^ leading to an amide bond formation. The peak of the C=O group for MWCNT gets shifted or is almost overlapped due to amide bond formation at same place. From these results it can be clearly said that poly-l-lysine gets covalently grafted to both MWCNT and GO sheets respectively and also that the GO is reduced to rGO state. The presence of free NH_3_^+^ groups is extremely useful for further application and functionalization of our rGO-MWCNT-PLL composite film.

**Figure 5:**
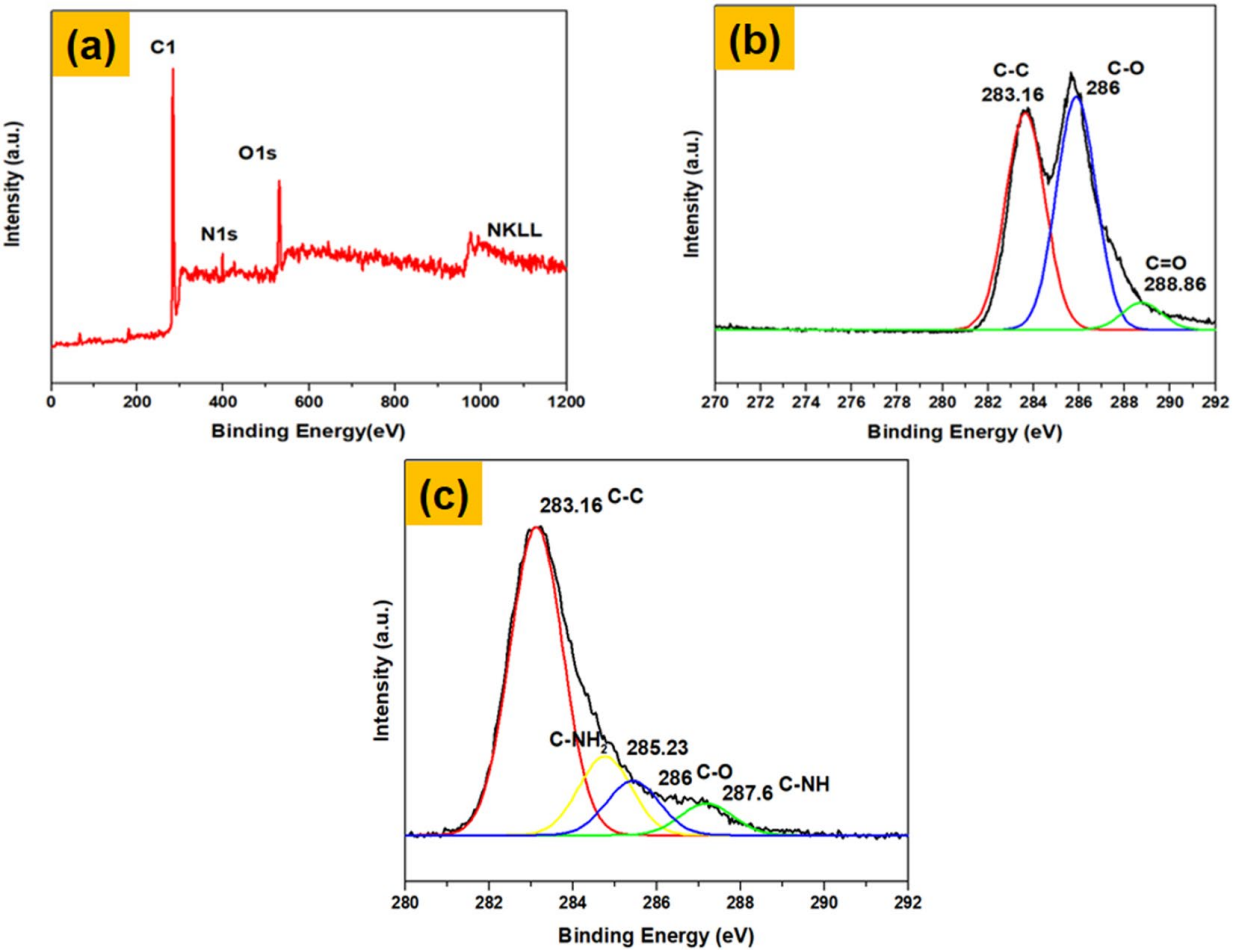
XPS spectra of full scan of the nanocomposite. (**a**) C1s of graphene oxide (**b**) and C1s of rGO-MWCNT-PLL nanocomposite(**c**).

The reduction of GO to rGO as well as the functionalization of PLL, upon MWCNT and rGO sheets was also further confirmed by XPS the full scanning spectrum is shown in Fig. 5a. In the rGO-MWCNT-PLL nano-composites (Fig. 5c) the XPS spectra showed a significant change and decrease in the intensity peak at 286 and 288.6 eV respectively as compared to the C1s peak of GO (Fig. 5b), which clearly indicates a loss of C-O and C=O functionalities. This is more so as there is a removal of the oxygen groups i.e. deoxygenation has occurred due to the decrease of carboxyl, epoxy and alkoxy groups^26^. Additionally, there are two distinct absorbance peaks which can be seen at 285.23 and 287.6 eV respectively due to the bond formation process which corresponds to the carbon in the C-NH_2_ and C-N bonds, respectively (Fig. 5c). This confirms with our FTIR result discussed above.

The atomic weight percentage of C1s, O1s and N1s as obtained from XPS is mentioned in Table 1. In Table 2, the percentage content of C1s (chemical states) is shown for rGO-MWCNT-PLL nanocomposite. In our previously reported article^26^ the percentage of C-O is higher (33.90), in graphene oxide compared to rGO-MWCNT-PLL nanocomposite due to the presence of epoxy, alkoxy, carbonyl and carboxyl groups on the surface of graphene oxide. The percentage content of C-C is more in rGO-MWCNT-PLL nanocomposite, but in rest of the bonds it has decreased compared to GO^26^. This changes in the nanocomposite confirms the formation of our product, by reduction of GO to rGO and decrease of the carboxyl and epoxy groups.

**Table 1:** The atomic percentage of elemental composition of rGO-MWCNT-PLL nanocomposite.

Material	Atomic Percentage of Elemental Composite		
	C1s	O1s	N1s
rGO-MWCNT-PLL	82.8	14.8	2.3

**Table 2:** The chemical states of C1s(%) of rGO-MWCNT-PLL nanocomposite.

Material	The Chemical states of C1s(%)		
	C-C	C-O	C-NH
rGO-MWCNT-PLL	76.42	17.76	5.94

Further confirmation about the formation of the nano-composites which is confirmed by the UV-visible spectroscopy shown in supplementary info Fig. S1. The GO-MWCNT composite show a peaking at 284 nm, while the nanocomposite show a peaking at 273 nm. The shifting in peak has been observed possibly due to the changes in electronic configuration in GO sheets and also due to the interaction of the GO-MWCNT composite with the PLL leading to reduction of the exposed GO sheets shown in Fig. S1^21^. Further confirmation about formation of the composite is also investigated through FESEM, HRTEM and AFM. The presence of the elements have been further confirmed through EDAX mapping.

**Figure 6:**
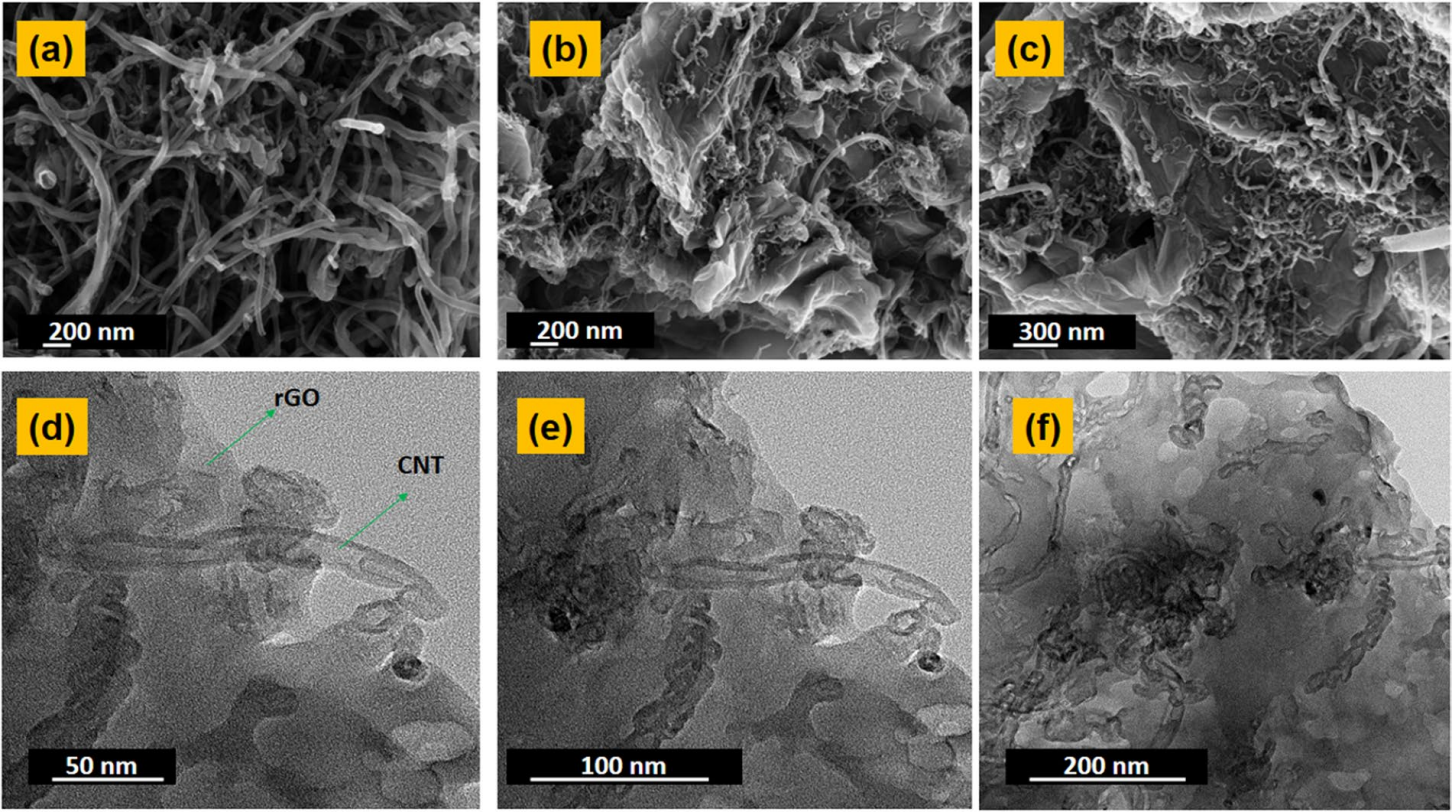
(**a**) FESEM images of bunch of CNTs. (**b**,**c**) Images of CNTs scattered on rGO sheets. (**d**–**f**) HRTEM images of CNTs attached on the rGO sheets at different magnifications.

It can be clearly observed from Fig. 6(a–c) that there are hollow rod like shaped bunches of MWCNTS that are attached on the surface of rGO sheets. It can be intuitively understood that in this configuration there may be an obvious prevention of any further agglomeration of rGO sheets. Due to prevention of agglomeration there is an exposure of a large unpiled surface area with more number of free amine groups hanging directly and freely of the nanotube bundles as well as on the 2-D sheets thus leading to more number of attachment sites for the enzymes. This leads to an overall faster response and lower limit of detection for our cantilever devices which is coated with the films.

The rGO-MWCNT-PLL composites were further studied using TEM and HRTEM Fig. 6(d–f) at different magnifications. It can be clearly observed that the size of the translucent rGO sheets are quite large. The CNTS are also large in length spreading all over the rGO sheets as bunches. The CNTS were also attached and evenly distributed in between the layers of rGO sheets forming a 3-D distribution which also prevented the stacking of rGO layers.

**Figure 7:**
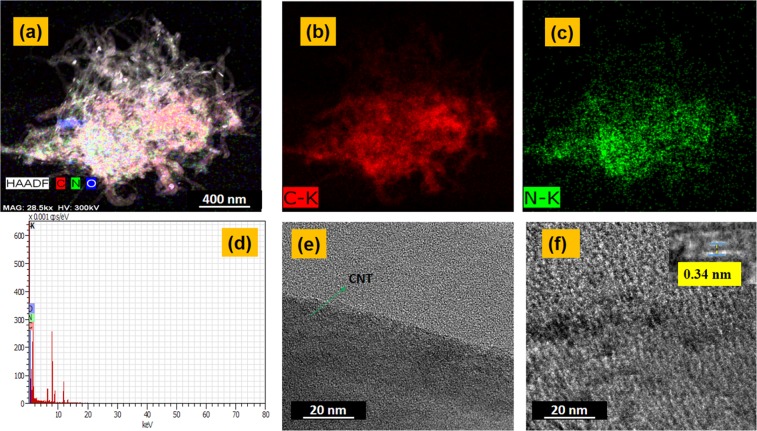
(**a**) HAADF-STEM image of rGO-MWCNT-PLL (**b,c**) elemental mapping of C and N. (**d**) EDX mapping. (**e,f**) High resolution image of rGO-MWCNT and MWCNT.

The elemental mapping of the nano-composite were further characterized through EDAX shown in Fig. 7(a–d). The presence of red and green dots at the same spatial location of carbon and nitrogen atoms points to the formation of multiple amide bonds as well as the presence of free hanging amine groups on the CNTs and rGO sheets. From these EDAX maps the formation and existence of the nano-composite is predicted. Figure 7(e) shows the interface or the boundary of CNT and rGO sheets. It can be clearly observed the CNT are adhering to the surface of the rGO sheets at a higher magnification. The inset in the Fig. 7f shows the lattice fringes with a spacing of d = 0.34 nm on the CNT layer corresponding to the distance between two single layers of Graphene sheets^27^.

**Figure 8:**
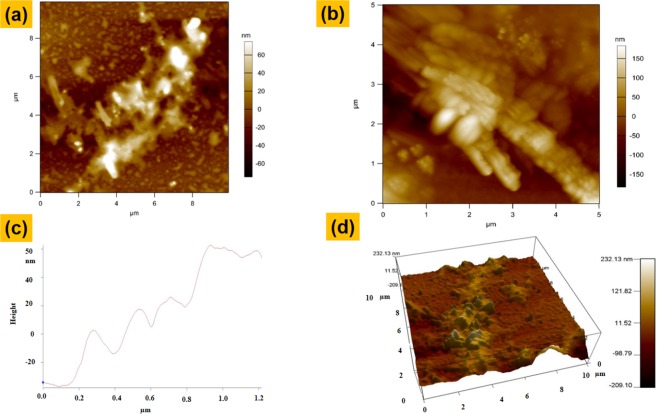
(**a**) Top view of rGO-MWCNT-PLL composite at 8 μm × 8 μm scanning area. (**b**) MWCNT bunch at higher magnification. (**c**) Graph obtained from line scanning above the layers of rGO-MWCNT-PLL composite sheets, showing the depth or thickness of individual layers d.3D view of rGO-MWCNT-PLL composite.

To further study the morphology of the material and also to know about the exact position of the inter-layers an AFM scan is carried out on the rGO-MWCNT-PLL composite films. The Fig. 8a shows the top view of the composite (10 μm × 10 μm) showing the rGO sheets with CNTs attached on it. The second Fig. 8b shows a magnified image with a scanning area of 5 μm × 5 μm. It can be clearly observed that the bunch of CNTs are present on the surface of the r-GO sheets more like a bush type of vertically aligned structure. In the third Fig. 8c graph obtained from line scanning above the layers of rGO-MWCNT-PLL composite sheets, showing the depth or thickness of individual layers, the rGO layer has been observed at a height of 40 nm. The second peak observed at 60 nm is due to attachment of MWCNTs to the rGO sheets. The third peak is most probably due to the poly-l-lysine attachment on the CNTs because it is almost at the same height of CNTs with an increase in height of only around 5 nm. The fourth peak was observed due to the second layer of rGO sheets. In the last Fig. 8d we observe the 3-D view of rGO sheets with CNTs and PLL attached on it^21^.

### SU-8 Cantilever Fabrication and immobilization of rGO-MWCNT-PLL nano-composites on the surface of the cantilever

**Figure 9:**
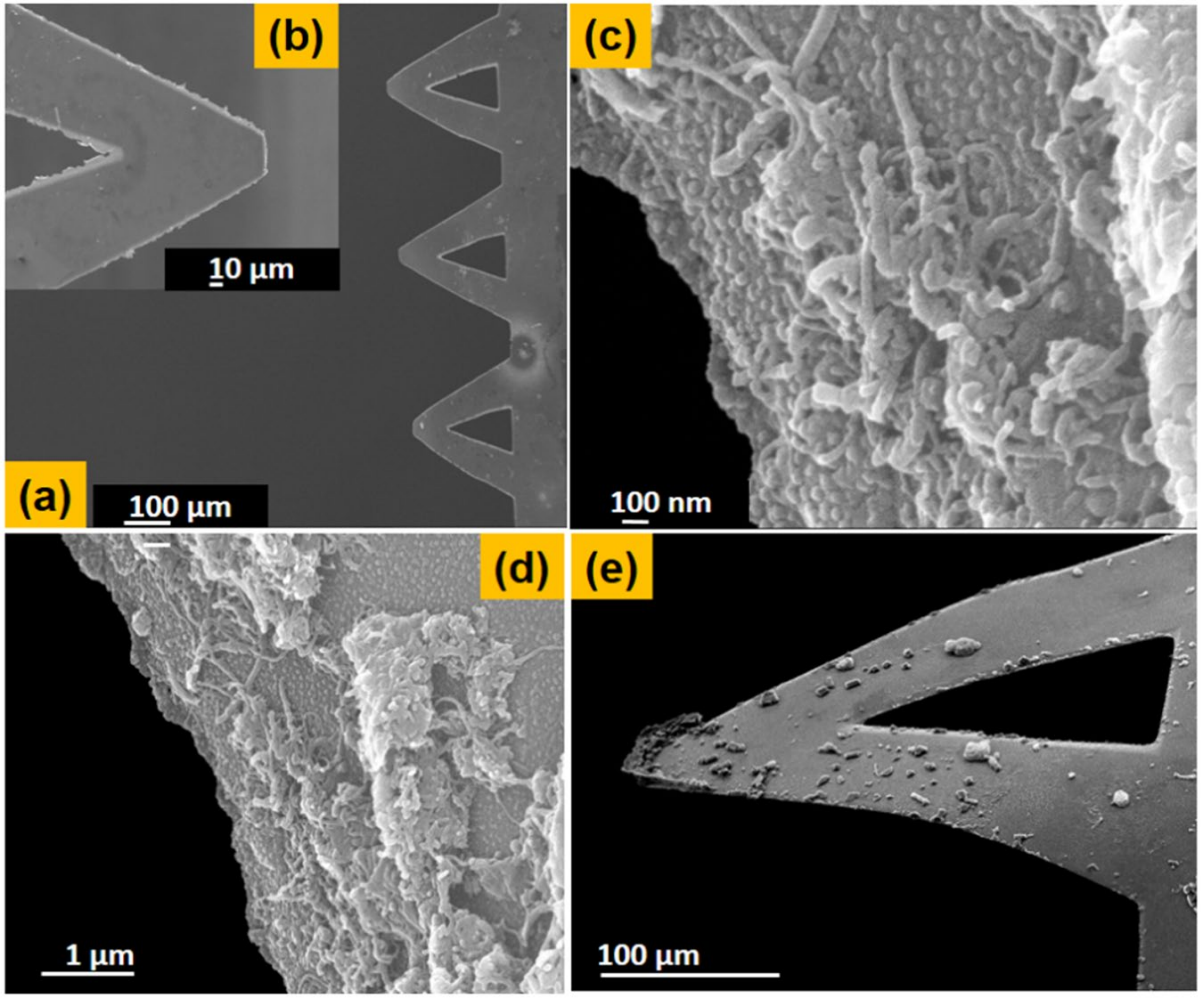
(**a**,**b**) FESEM images of an array of V-shaped cantilever and magnified image of a single cantilever. (**c**,**d**) Shows high magnified and low magnified images of rGO-MWCNT-PLL nanocomposite attached on the SU-8 cantilever surface. (**e**) Shows the bent image of the cantilever.

SU-8 micro-cantilevers were fabricated using a simple two-step process already discussed in methods. For further details related to the fabrication of the cantilever one can find the same in supplementary info Fig. S2. Cleaned SU-8 cantilevers are plasma treated at a low power to make the surface highly hydrophilic due to the surface modification and increase in the surface hydroxyl groups. After that the cantilevers are covered with rGO-MWCNT-PLL nano-composites of 0.5 mg/ml for 30 mins followed by washing with PBS buffer. The cantilever chips are then soaked and dried overnight with 1 mg/ml cholesterol oxidase (highly selective material)^28,29^ at 4 °C. For the sake of comparison the cantilevers are treated with MWCNT-PLL, GO-PLL and only PLL with same procedure. Figure 9(a,b) show the FESEM images of an array of V-shaped cantilevers and the inset Fig. 9(b) shows a magnified image of a single cantilever. The Fig. 9(c,d) show high resolution images of rGO-MWCNT-PLL nanocomposite film as attached on the modified SU-8 cantilever surface. Figure 9e shows another image of the cantilever after bending as the interaction of cholesterol oxidase with cholesterol takes place.

### Study of Fluorescence of FITC tagged rGO-MWCNT-PLL composite and RITC tagged Cholesterol oxidase enzyme

**Figure 10:**
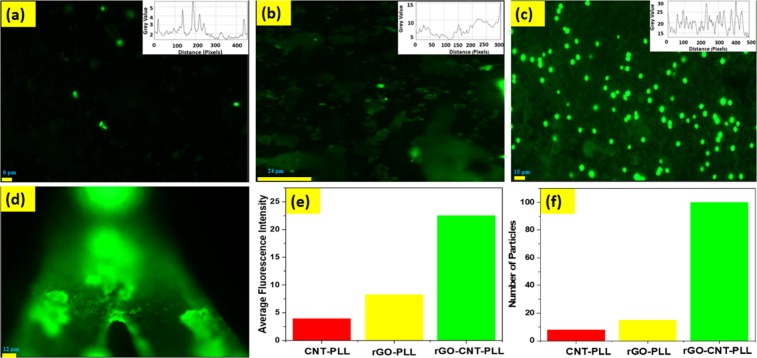
(**a**) Show the FITC tagged MWCNT-PLL composite upon SU-8 surface at 30x magnification. (**b**) Shows the FITC tagged rGO-PLL composite upon SU-8 at 30x magnification (**c**) Shows the FITC tagged rGO-MWCNT-PLL composite upon SU-8 at 30x magnification. (**d**) Shows the FITC tagged rGO-MWCNT-PLL coated SU-8 cantilever at 50x magnification. (**e**) Shows the comparison of average of fluorescence intensity for the three nanocomposites. (**f**) Shows the comparison of number of particles immobilized upon SU-8 surface

The images below show the FITC tagged MWCNT-PLL, rGO-PLL and rGO-MWCNT-PLL composites. The inset of Fig. 10(a–c) show plots of the grey values obtained from fluorescent images using image J software with respect to distance across the frame in pixels. Of significant mention are Fig. 10(c,d) showing the FITC tagged rGO-MWCNT-PLL coated cantilever surfaces are magnifications of 30X and 50X respectively. The Fig. 10e shows the comparison of average fluorescence intensity obtained from images for the three nanocomposites. It was observed the rGO-MWCNT-PLL showed highest no. of fluorescent plots as compared to other composites. It was also observed the rGO-MWCNT-PLL nano-composites showed maximum immobilization in terms of site densities. The number of nanoparticles attached to the SU-8 surface after washing with buffer was highest in case of the newly formed nano-composites shown in Fig. 10f. The higher fluorescence and higher number of particles prove the existence of more number of free amine groups on the surface of the films for sensing.

**Figure 11:**
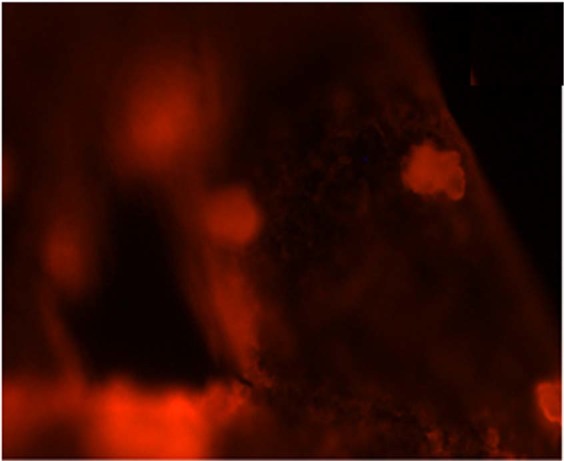
Shows the RITC tagged cholesterol oxidase coated SU-8 cantilever at 50x magnification.

To further know about the presence of the surface immobilized Cholesterol oxidase on the top of PLL functionalized rGO-MWCNT films, a separate fluorescence based study is performed with Rhodamine B isothiocyanate (M/s Sigma Aldrich) (RITC) dye. A protocol similar to that of followed FITC tagging detailed in the methods section in main manuscript was deployed on surfaces of the micro-cantilevers. The red color fluorescence can be observed on the immobilized cantilever surface at 50 X magnification Fig. 11. This shows that there is partial binding on cantilever surface according to the reach of the RITC labeled cholesterol oxidase. As the liquid film evaporates (application method being drop casting) we can see the concentration of the RITC labeled species along evaporated boundaries although the overall binding with the film is being reflected by the red presence over the whole surface in lower intensities. As the imaging of the cantilever is carried out post washing it provides a very firm evidence of immobilization of RITC labeled Cholesterol oxidase molecules over the whole cantilever surface.

### Comparative study of deflection of cantilever with immobilized films

**Figure 12:**
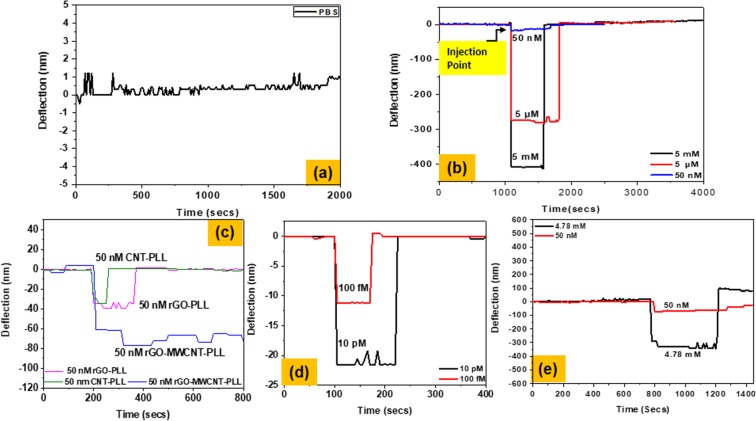
(**a**) Deflection of the cantilever when only PBS flown through the flowcell (oscillations are observed due to thermal noise and their magnitude is only 1~1.5 nm). (**b**) Deflection of the PLL coated cantilever when cholesterol of different concentrations 5 mM, 5 μM and 50 nM flown through the flowcell. (**c**) Comparison of deflection of the MWCNT-PLL, rGO-PLL, rGO-MWCNT-PLL coated cantilever at 50 nM concentrations of cholesterol. (**d**) Deflection of cantilever at 10 pM and 100 fM concentration of cholesterol. (**e**) Response of the cantilever when blood sample flown through the flowcell.

Cantilevers immobilized with the various films are next placed in a flow cell-Chamber volume 100 µl made of acrylic. The flow rate into this chamber is maintained at about 5 μl/min operated through a dual syringe pump (New Era Pump Systems, Inc). At the beginning the deflection reading is taken by flowing phosphate buffer solution (pH 7) in the chamber where the cantilevers are withheld. The fluctuations or deflection obtained are recorded to be too small in the range +1 nm [See Fig. 12a]. In the next step two laser spots are focused on the two cantilevers, one simply PLL coated and the second one being uncoated SU-8 cantilever. Cholesterol is (M/s SRL pvt Ltd., CAS no. 57-88-5) diluted at various concentrations using phosphate buffer. Deflection measurements are performed corresponding to several different dilutions (viz., cholesterol concentrations of 5 mmol, 5 µmol and 50 nmol) [Fig. 12b]. They are seen to be significant. At the 50 n mol concentration the deflections are too small and are not so distinctive although after the surface attachment of MWCNT-PLL, rGO-PLL and rGO-MWCNT-PLL composites the deflections get significantly altered. The cantilever shows double deflection height in case of rGO-MWCNT-PLL coated composite compared to the rGO-PLL and MWCNT-PLL [See Fig. 12c] which confirms the high density of the immobilization sites on the new composite film. Thee sensitivity thus gets significantly altered by using the new composite and a LOD upto 100 femtomolar concentration is seen [See Fig. 12d]. The response and recovery times are calculated from the baseline (injection point) upto the saturation point (plateau). The response time of the cantilever with PLL functionalization at 50 nmol concentration is 16 secs whereas the MWCNT-PLL coated cantilever shows a response time of 13 secs and the rGO-PLL coated cantilever has a response time of 12 secs. In case of the newly developed rGO-MWCNT-PLL film the cantilever response comes out as 9 secs, presuming that the changes in signal do not have any inertial delays. Similarly, the response at 10 pM and 100 fM concentration is 5 secs and 4 secs only for rGO-MWCNT-PLL coated cantilever. While there may be a possibility of changes surface stiffness of the cantilever but as the films are all quite thin with respect to the SU-8 cantilever thickness such a change in the mechanical property of the films may not significantly alter the compounded stiffness. The cantilevers were washed using buffer by flowing PBS solution into a flow-cell where the micro-cantilevers were positioned. Further the wash step was carried out using constant flow-rate (2 micro-liter/min.). We observe 100% recovery of the cantilever sensors in Fig. 12(b,d). In case of rGO-MWCNT-PLL coated cantilevers as reported in Fig. 12(c) it is seen that the cantilever oscillates around −70 nm permanent deflection without showing full recovery of deflection. We have already seen using scanning and transmission electron micrographs etc. that our cantilevers having rGO-MWCNT-PLL composites are 3-D coated with multi-layers of PLL functionalized sites along the thickness of the films with interlayer MWCNTs. We hypothesize that this may make the structure more amenable to analyte diffusion until the surface is saturated. While doing the wash cycle, as the release of bound species from the surface is mostly accomplished by a pH change it may be possible that the pH change may not happen so well in the bulk 3-D structure of rGO-MWCNT-PLL composites as opposed to pure 2-D surfaces of CNT-PLL or rGO-PLL films. As more of bound molecules in the bulk are left over after a similarly timed wash step in the rGO-MWCNT-PLL composites films their recovery may not be 100%. Subsequently in Fig. 12d,e there is an indirect proof of this hypothesis as at lower concentrations of cholesterol in the picomolar or subsequently femtomolar range the cantilevers are found to perfectly recover. A greater penetration depth of the analyte is therefore imminent in the higher concentration cases (50 nm case) in the films. Thus we can say that our sensors are tunes to low concentration detection with high repeatability.

Detection on the cantilevers are also done from human blood samples. Blood samples of about 5 ml are collected and centrifuged at 4000 rpm. The serum is next separated from the blood sample is investigated for cholesterol. Any significant deflection of the cantilever was observed corresponding to 5 m molar concentration of cholesterol. The clinically obtained result showed 4.78 mM concentration. The blood samples were further diluted in nano-molar concentration using buffer solution maintaining pH 7 and were tested on cholesterol oxidase coated rGO-MWCNT-PLL composite films. The cantilevers showed higher sensitivity due to more number of molecular adsorption sites and better response time of only 7–8 secs [See Fig. 12e]. There is an observation in Fig. 12(e) where corresponding to an extracted serum sample from whole blood (which corresponds to 4.78 mM” as observed with electroluminescence in a pathology lab) the cantilever response shows an upward differential causing a reverse bending away from the baseline. The observation being done with blood serum containing the above concentration of cholesterol also carried many non specific proteins along with the analyte. It is very much possible that there were proteins in the sample which may be absorbed on both surfaces of the cantilever where washing may not be that efficacious. Thus there is an upward differential created in the trial carried out whereas actually in the other cases the wash step was able to get rid of the adsorbed cholesterol from the bottom surface of the cantilever completely. Below Table 3 shows deflections obtained for the different composites at different concentrations.

**Table 3:** Deflection obtained at different concentrations of cholesterol with different composites.

Material	Concentration	Deflection (nm)
PLL	5 m molar	403
PLL	5 µ molar	273
PLL	50 n molar	16
rGO-PLL	50 n molar	39
MWCNT-PLL	50 n molar	33
rGO-MWCNT-PLL	50 n molar	80
rGO-MWCNT-PLL	10 p molar	22
rGO-MWCNT-PLL	100 femto molar	12

### Selectivity of the sensor

Selectivity is an important parameter to examine the performance of any sensor. To study the selectivity of our sensor in detection of cholesterol, the sensor was tested with 50 nm concentration of L-ascorbic acid, glucose and uric acid. The deflections recorded for this analyte was within 10 nm, whereas for cholesterol at same concentration the deflection was around 80 nm. It means that the deflections obtained for cholesterol show 8 times more responsivity as compared to other analytes. It can be clearly interpreted behavior that the cholesterol oxidase coated cantilever sensors are highly selective for cholesterol catalysis as shown in supplementary Fig. S3.

## Conclusions

rGO-MWCNT-PLL composite films have been synthesized are evaluated for bindability to functional groups. Cholesterol was used as an analyte of detection although the high efficacy of binding process over these new films give way to enhance sensitivity of SU-8 microcantilevers. Cholesterol levels in phosphate buffer upto a limit of 100 femto mol of order is detected by microcantilevers coated with rGO-MWCNT-PLL films. The cantilevers have also shown an overall faster response and greater deflection when coated with the nanocomposites as compared to MWCNT-PLL, rGO-PLL and PLL coated SU-8 cantilever. The detection of cholesterol carried out with serum extracted from whole blood indicate that the nanocomposite synthesized has a major role to enhanced sensitivity as well reducing time of response. Thus certainly the novel films developed in this work may provide a potential immobilization mechanism to bind and detect proteins sensitivity over cantilever bio-sensors.

## Methods

### Preparation of GO sheets and rGO thin film based composite

GO sheets were prepared by exfoliation of graphite flakes [M/s Loba Chemicals pvt ltd.] by Modified Hummers Method^30–32^. 1.5 mg of GO powder synthesized by modified Hummer’s method was dispersed along with 1.5 mg of carboxylated MWCNT (obtained through acid treatment), 2 mg of N,N′-Dicyclohexylcarbodiimide (DCC) [Spectrochem Chemicals pvt ltd.] and 6 mg of Poly-l-lysine Hydrobromide[Sigma Aldrich] were all dispersed in dimethyl formamide (DMF) and was magnetically stirred vigorously for 24 hrs at a temperature of 50 °C. This mixture was centrifuged using ethanol and milli-q-water repeatedly (3–4 times) so that the excess DCC and PLL could be removed from the composite. The mixture was then dried in a hotplate at 50 °C for about 2 hrs to formulate a residue that was the functionalized composite rGO-MWCNT-PLL shown in Fig. 1. Graphene oxide gets reduced to reduced graphene oxide, as observed in the characterization results.

Experiments with human blood sample were performed in compliance with the national guidelines and relevant laws (Ethical Guidelines for Biomedical Research on Human Participants, provided by Indian Council of Medical Research), and the ethical clearance for the experiments provided by the GSVM Medical College, Kanpur.

### Material Characterization

The crystalline structure of the as obtained GO was characterized using XRD (M/S Panalytical Xpert ProMPD system) with a Cu target (λ = 1.540598 Å) and a scan rate of 3°/min as reported in our earlier work^26^. Raman spectroscopy was also done for the GO and the rGO composite film. FTIR spectra was further recorded by Kbr pellet method. UV-Vis spectroscopy measurements were performed on the rGO composite films using DMF dispersant. Morphology of the drop casted composite films were analysed through FEI Titan G2 60–300 TEM (HRTEM) and FESEM (M/S Oxford instruments) as well as AFM (M/S oxford instruments Asylum Research) were both carried on the composite films.

### Fabrication of Cantilever

SU-8 Cantilevers upto 250 nm thickness were fabricated using SU-8 2000.5 on another base layer that was formulated by lithography and pattering using SU-8 2035 on SiO_2_ substrates. The details of fabrication have been reported in an earlier work and the fabrication flow chart for the same can be obtained in supplementary information Fig. S2.The cantilevers were finally released from the substrate by an etching step used on SiO_2_ by using Buffer HF (BHF)^31,32^.

### Printing of the composite films on cantilever surfaces

The Cantilevers as obtained as per details earlier were firstly plasma treated using low power plasma and then coated using drop casting method through a nano-molar dispersion of the composite powder in Dimethyl Formamide (M/s Loba Chemicals). The dispersion is first ultrasonicated for 30 mins and dispensed using a 5 micro-liter Hamilton syringe (M/s Sigma Aldrich). The syringe was held in a clamp on a XYZ stage for precise positioning of the syringe over the cantilever under an optical microscope before dispensing the solution over the cantilever surface. The coated surfaces of the cantilevers were completely room dried overnight. After the solution was fully dried on the surfaces of the cantilevers and it formulated a thin film, the cantilevers were coated using cholesterol oxidase which was freeze dried overnight at 4 °C temperature. Surface coverage of the nano-composite films on the cantilever surfaces analyzed using Nikon Microscope at high magnification upto 50x and FESEM. Here, four different cantilevers were coated, one with only PLL, one with CNT-PLL composite, another with PLL functionalized GO layers and yet another with rGO-MWCNT-PLL composite solution. While coating the cantilevers with various solutions very minute deflections were observed but as they stabilized all of them returned to equilibrium position and maintained slightly deflected positions with some degree of surface stress induced due to each immobilization step. Their positions were used to baseline the four independent cantilevers and any further deflections due to the immobilization of the target analyte started off with respect to this slightly pre-stressed condition. The process of drop casting was carried out precisely under a Microscope aperture that and the plunger of the micro-syringe was very carefully pressed to dispense slowly without spillage from the surface to surroundings.

### FITC tagged nanocomposites for immobilisation

0.5 mg of MWCNT-PLL, rGO-PLL and rGO-MWCNT-PLL were separately dispersed in 1 ml milli-Q-water each. The composites were ultra-sonicated for 45 mins, to obtain uniform dispersion. A total volume of 10 µl solution of each type was drop-casted using Hamilton syringes on the surfaces of different SU-8 surfaces. 100 µg/ml, FITC solution was prepared in carbonate/bicarbonate buffer at pH 10.5. For labeling of the coated films, around 5 µl of FITC solution was poured over each surface and incubated for 30 mins at 37 °C. The samples were then washed thrice with the buffer solution to remove unbound dye molecules following which they were mounted on glass slides and imaged with a fluorescent microscope as shown in Fig. 10(a–c). Another parallel trial of the rGO-MWCNT-PLL film was made on a SU-8 Micro-cantilever surface with a similar labeling procedure. The FITC molecules actually bind through amide linkages with all the three nanocomposites films. The gradual increase of the fluorescence intensity over the MWCNT-PLL, rGO-PLL and rGO-MWCNT-PLL films respectively as captured in Fig. 10(a–c) supports the claim of the amide binding in a three dimeniosnal manner to the rGO as well as MWCNTS. A similar behavior is seen over the cantilevers which means that nanocomposites rGO-MWCNT-PLL is successfully functionalized with many binding sites. Similar, protocols were followed to know the presence of cholesterol oxidase upon cantilever surface by tagging with RITC using same buffer and maintaining pH.

## Supplementary information

Poly-L-Lysine functionalised MWCNT-rGO nanosheets based 3-d hybrid structure for femtomolar level cholesterol detection using cantilever based sensing platform
