# Supplementary material for: Poly-L-Lysine functionalised MWCNT-rGO nanosheets based 3-d hybrid structure for femtomolar level cholesterol detection using cantilever based sensing platform
Source: Sci Rep. 2019 Mar 6;9:3686. doi: 10.1038/s41598-019-40259-5 (PMC6403341; doi:10.1038/s41598-019-40259-5)
Supplement: Supplementary file 1 — Poly-L-Lysine functionalised MWCNT-rGO nanosheets based 3-d hybrid structure for femtomolar level cholesterol detection using cantilever based sensing platform [file 41598_2019_40259_MOESM1_ESM.docx]

**Supplementary Information**

**Poly-L-Lysine functionalised MWCNT-rGO nanosheets based 3-d hybrid structure for femtomolar level cholesterol detection using cantilever based sensing platform**

Aviru Kumar Basu^1,2,3^, Amar Nath Sah^3,4^, Asima Pradhan^3,5^, Shantanu Bhattacharya^1,2^

1. Design programme, Indian Institute of Technology, Kanpur, U.P.208016, India

2. Microsystems Fabrication Laboratory, Department of Mechanical Engineering, Indian Institute of Technology, Kanpur, U.P.208016, India

3. Biophotonics Laboratory, Department of Physics, Indian Institute of Technology, Kanpur, U.P.208016, India

4. Department of Biological Sciences and Bioengineering, Indian Institute of Technology, Kanpur, U.P.208016, India

5. Department of Physics, Indian Institute of Technology, Kanpur, U.P.208016, India

#corresponding author: [bhattacs@iitk.ac.in](mailto:bhattacs@iitk.ac.in)


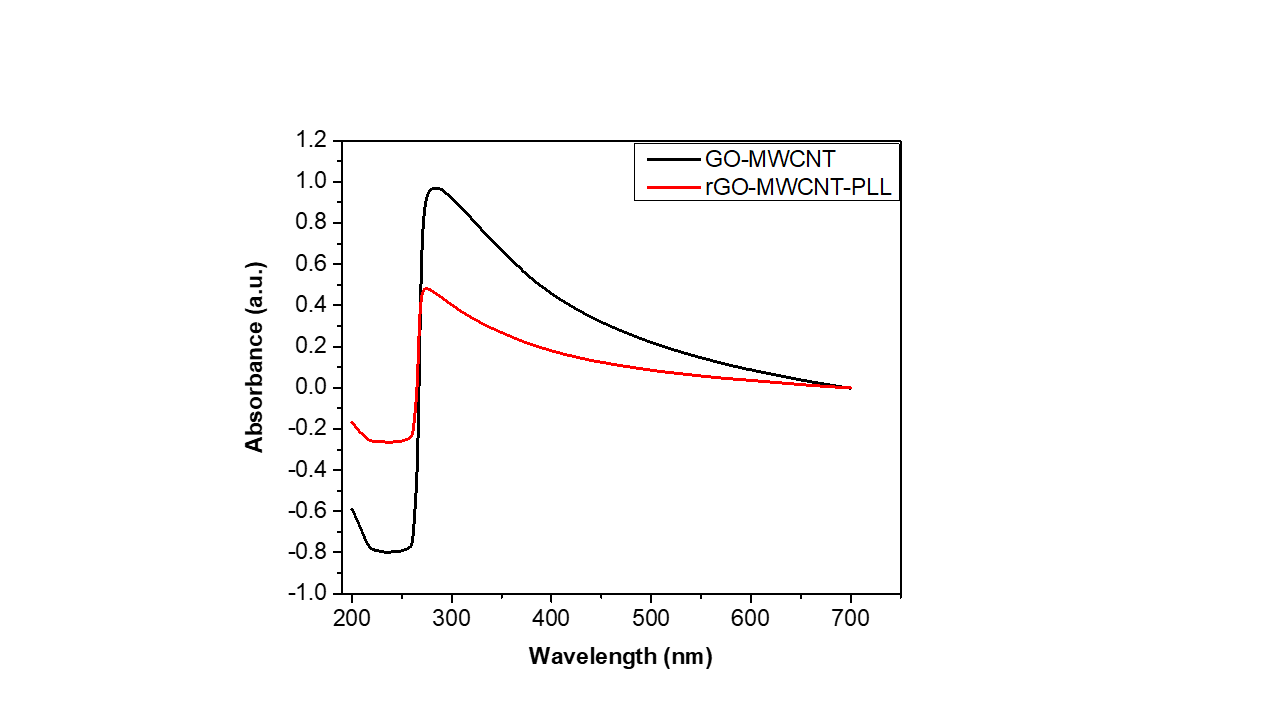


**Fig.S1** UV-VIS spectra of GO-MWCNT and rGO-MWCNT-PLL composite


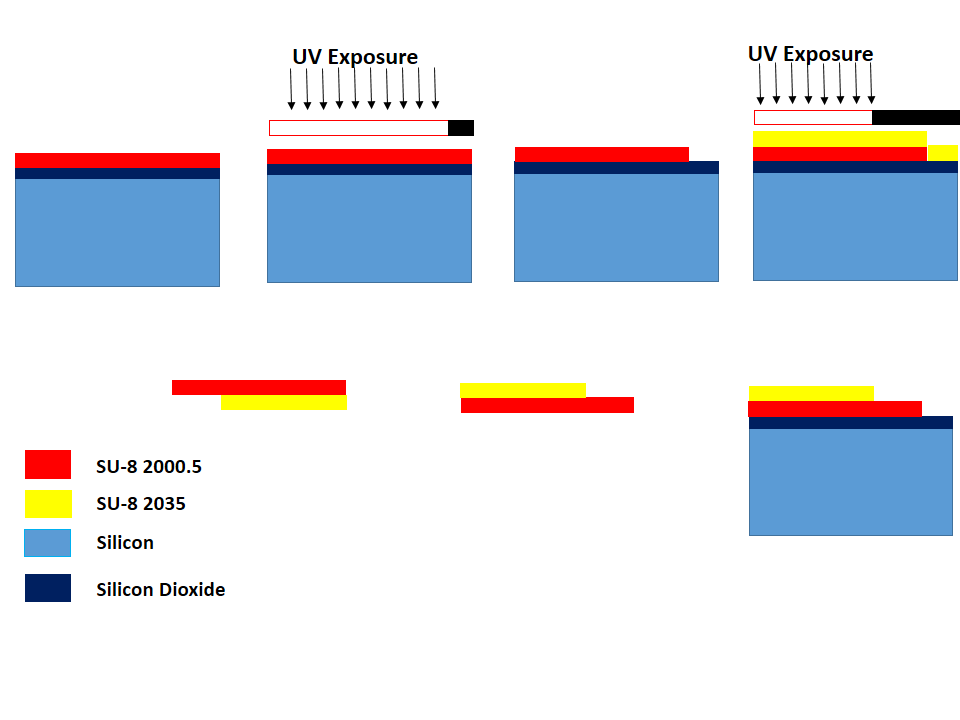
 **Fig.S2** Schematic of Fabrication of Cantilever


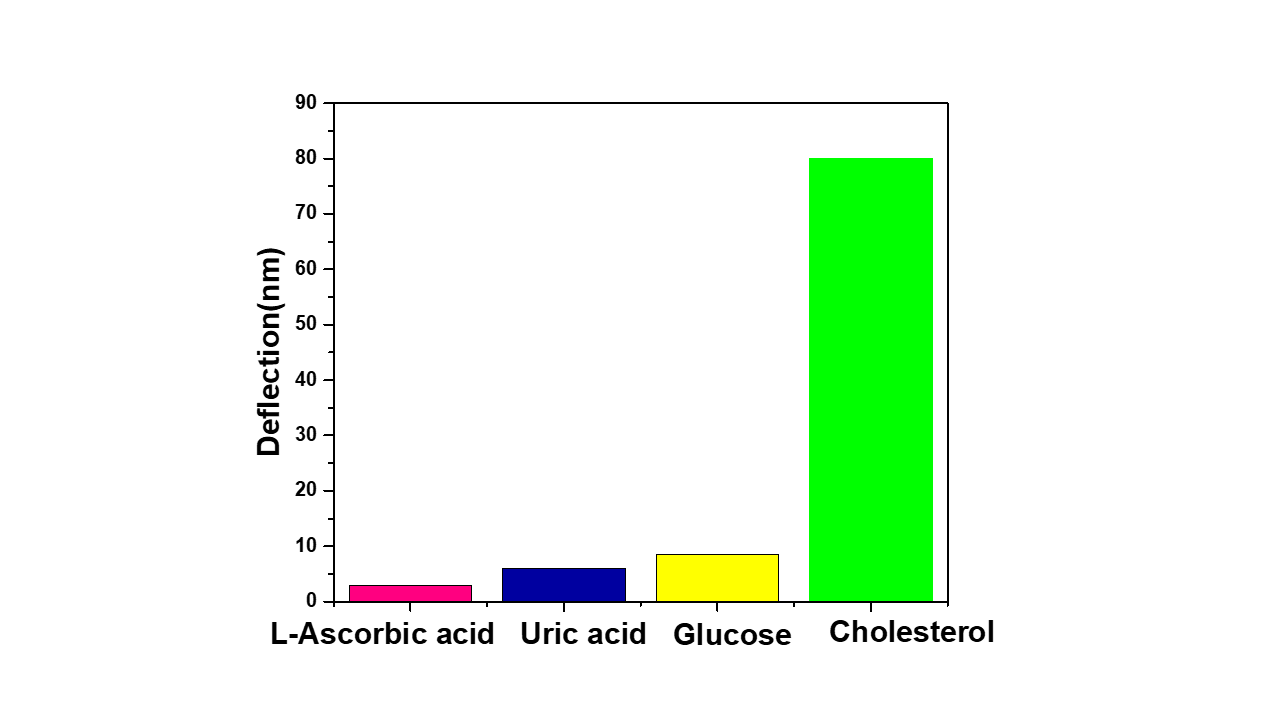


**Fig.S3** Deflection obtained for all the analytes present at 50 nM concentration (selectivity curve).
